# Lending a Helping Hand at Work: A Multilevel Investigation of Prosocial Motivation, Inclusive Climate and Inclusive Behavior

**DOI:** 10.1007/s10926-016-9680-z

**Published:** 2016-11-11

**Authors:** Philippe T. J. H. Nelissen, Ute R. Hülsheger, Gemma M. C. van Ruitenbeek, Fred R. H. Zijlstra

**Affiliations:** Department of Work and Social Psychology, Maastricht University, P.O. Box 616, 6200 MD Maastricht, The Netherlands

**Keywords:** Inclusive climate, Inclusive (helping) behavior, Prosocial motivation, People with disabilities, Workplace inclusion, Multilevel modeling

## Abstract

*Purpose* People with disabilities often encounter difficulties at the workplace such as exclusion or unfair treatment. Researchers have therefore pointed to the need to focus on behavior that fosters inclusion as well as variables that are antecedents of such ‘inclusive behavior’. Therefore the purpose of this study was to research the relationship between prosocial motivation, team inclusive climate and employee inclusive behavior. *Method* A survey was conducted among a sample of 282 paired employees and colleagues, which were nested in 84 teams. Employees self-rated prosocial motivation and team inclusive climate, their inclusive behavior was assessed by colleagues. Hypotheses were tested using multilevel random coefficient modeling. *Results* Employees who are prosocially motivated will display more inclusive behavior towards people with disabilities, and this relationship is moderated by team inclusive climate in such a way that the relationship is stronger when the inclusive climate is high. *Conclusion* This study shows that inclusive organizations, which value a diverse workforce, need to be aware of not only individual employee characteristics, but also team level climate to ensure the smooth integrations of people with disabilities into regular work teams.

## Introduction

**Fig. 1:**
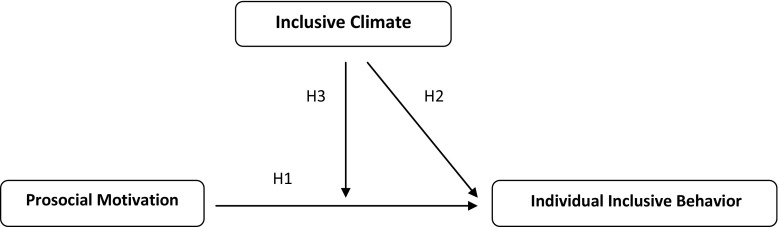
Multilevel and cross-level processes of inclusive behavior

In an ever changing European society that is currently discerning both the rising number of baby-boom generation retirees and a diminishing labor force, there is a need to focus on employing disadvantaged groups, such as people with disabilities. Not only to embrace people with disabilities in working society or to counter their low employment rates, but also to allow the social security system to be upheld [1, 2]. Therefore, the European Commission stimulates the participation of people with (physical and non-physical) disabilities to the labor market in their 2011–2014 strategy by stressing that corporate social responsibility is beneficial to both enterprises and the society as a whole [3, 4]. In the US, legislation such as the ADA (Americans with Disabilities Act 1990) and the ADAAA (ADA Amendments Act 2008) were devised to attain similar goals and protect people with disabilities from employment discrimination. However, employment issues and biases (e.g. disclosure decisions, low performance expectations, stereotyping, or limited growth opportunities) persist for people with disabilities once they have entered the labor market [5, 6]. These problems often originate from exclusion and unfair treatment by their work colleagues [7]. To address such issues there is a need to learn more about how organizations can facilitate inclusion. Yet, research on how inclusion in organizations can be fostered remains scarce, and Industrial and Organizational (IO) research is requested to focus on factors that enable the accommodation of people with disabilities at the workplace [5, 6]. With the present study we follow this call by studying factors that contribute to inclusion both at the individual- and the team-level of analysis. Specifically, we build upon the prosocial motivation and the team climate literatures and suggest that individual prosocial motivation and team inclusive climate both contribute to foster inclusion at work. As an outcome variable, we focus on *inclusive behavior*, which we conceptualize as a form of citizenship or helping behavior that is specifically directed at coworkers with disabilities [8]. Figure 1 depicts the corresponding model.

## Inclusive Behavior

Helping behavior at the workplace, in general, has received a lot of research attention in various forms such as (a) organizational citizenship behavior (OCB) [9, 10], (b) prosocial behavior [11–13], (c) citizenship performance [14], and (d) volunteer activities [15, 16]. These helping behaviors are clearly related concepts that have an important conceptual overlap since all of them refer to extra role behaviors which are volitional, discretionary and intended to benefit others [13, 14, 17]. Organizational citizenship behavior reflects behavior that goes above and beyond the job description, and can be defined as “performance that supports the social and psychological environment in which task performance takes place” [18, p. 95] Most importantly, studies on helping behavior have made important contributions by revealing the beneficial consequences of helping on an individual, team, and organizational level, such as increase of employee performance and productivity [19], but also employee well-being [20]. Similarly, the present study aims at extending the line of prosocially motivated helping research by focusing on a specific kind of citizenship behavior that is tailored towards employees with disabilities, which we label inclusive behavior [8]. We therefore define inclusive behavior as extra role behavior that is intended to benefit people with disabilities at work. Parallel to the concept of OCBI (organizational citizenship behavior which targets to benefit the individual, and therefore indirectly benefits the organization), it is set up to represent the courtesy and altruism dimension of OCB [19, 21–23]. The goal of inclusive behavior is to benefit colleagues with disabilities at work by means of providing help with a relevant problem at the workplace (altruism) or by preventing such work-related problems (courtesy).

## Prosocial Motivation and Inclusive Behavior

Motivation explains why individuals initiate, direct, and prolong certain work related actions in general [24, 25]. Work related actions that are specifically aimed at benefiting others, such as coworkers, are deemed to stem from prosocial motivation [25]. Prosocial motivation, in turn, originates from the desire to meet internally set goals, and to stay true to one’s identity [26]. Moreover, prosocial motivation is argued to have its roots in people’s prosocial dispositions, values, and motives [17, 27, 28]. Prosocial motives and motivation therefore bear some similarities in that both represent an active concern for the welfare of others [29], a need to be helpful, as well as a desire to build helpful relationships with others [17].

In order to identify the motives or values that bring employees to engage in citizenship behaviors, researchers have used a functional approach [9, 17, 30]. The functional approach to motivation aims to clarify why people decide to perform extra-role behaviors [31] and suggests that people will willingly engage in helping behavior because such actions meet their own needs and it allows them to reach set goals [17]. Motives and values will thus provide insight in the rationale of people’s actions [31]. As prosocial values capture individuals’ need to be helpful and a desire to build positive relationships with others [17], employees with prosocial values will thus be more inclined to engage in helping behavior in general, including inclusive behavior. Furthermore, employees who are prosocially motivated have the desire to perform beneficial actions for others, because they care about changing others’ lives for the better [32], and will therefore be more inclined to display prosocial and other helping behaviors [28]. A number of studies have provided empirical evidence for the relationship between prosocial motivation and different kinds of helping or prosocial behavior (e.g. [17, 27, 32]. Specifically, in organizations which aim and value a diverse workforce that includes people with disabilities, prosocially motivated employees will have apt opportunities to help others and display inclusive behavior.

Given the arguments presented above, we expect that prosocially motivated employees will be more inclined to go the extra mile by displaying more inclusive behavior than low prosocially motivated employees.

### **Hypothesis 1**

There is a positive relationship between prosocial motivation and individual inclusive behavior.

## The Role of Context

Although we expect to find an overall positive relationship between employees’ prosocial motivation and inclusive behavior, we expect that contextual factors influence the strength of this relationship. A number of studies have revealed a positive relationship between prosocial factors and various forms of helping behavior [11, 12]. Grant and Mayer [27], however, argued that future research should consider moderators in order to gain a more comprehensive understanding under which conditions prosocial motivation results in citizenship behavior. Looking into moderators at the individual level of analysis, Grant and Mayer [27] were able to show that impression management motives interact with prosocial motives in predicting citizenship behavior. Additionally, Maner and Gailliot [33] found that kinship influences the motivation-helping dyad, in such a way that motivation predicts helping behavior more strongly if participants are related. Other research illustrates that the relationship between prosocial motivation and prosocial behavior is influenced by the way jobs are designed [28, 34]: The prosocial motivation-helping behavior relationship was stronger when employees had the opportunity to witness the perceived beneficial consequences of their actions.

In the present study we build upon and extend these findings on individual-level moderators of the prosocial motivation - behavior link by investigating how contextual, team-level variables shape the prosocial motivation - inclusive behavior relationship. In recent years, researchers have increasingly started to focus on contextual variables of the work environment, investigating them not only as direct predictors of individual work behavior but also as moderators [35–38]. Since organizations are multilevel entities, it is important to take into account variables at more than one level [35], as considering contextual variables as moderators helps shedding light on relationships that might otherwise be overlooked [36]. In this way, a study on individual helping behavior found that group trust moderates the relationship between affective commitment and interpersonal helping behavior [35], such that employees are more likely to help others when interpersonal trust was high.

To continue on this new multilevel road, the goal of the present study is to go beyond the individual level and to shed light on team-level conditions that channel the relationship between prosocial motivation and inclusive behavior. Specifically, we introduce the concept of “inclusive climate” and investigate its role as a contextual variable on the relationship of prosocial motivation with inclusive behavior. In general, climate refers to the overall perceptions of the work environment at an aggregated or team level that represents the shared psychological meanings of a group [37, 39]. Colella and Bruyère [5] defined workplace inclusion as the degree to which “people with disabilities are accepted, helped, and treated as others by their coworkers” [5, pp. 492–493]. We look at inclusion at the team level of analysis and consequently define inclusive climate as team members’ norms and perceptions of the way people with disabilities are accepted, helped, and treated in their team. We build on literature on collectivistic norms [40] in arguing that inclusive climate has both a direct and an indirect influence on the amount of displayed inclusive behavior by individual team members.

When people form groups, norms are created to guide behavior [40]. As group norms are used as guidelines for employees to act within their social work setting, they will govern behavior according to the procedures set by the work group [40]. When being inclusive becomes the standard way to act in a group, employees will thus try to adhere to that norm in order to behave in a socially consistent way. In addition, specific group norms such as the norm to behave socially responsible might contribute to inclusive behavior because people attempt to preserve a positive view on themselves [13]. Furthermore, people who are focused on the collective, place high value on belonging to a group, and will therefore easily adhere to group norms to foster group well-being [41]. Even merely the prevalence of collectivistic norms may engage employees to express more prosocial motivation because norms dictate that group well-being is important [28]. Similarly, workers who adhere to collectivistic norms have been found to be related to within group helping behavior [42]. In all, collectivistic group norms, such as prevalent in a positive inclusive climate, might be positively related to inclusive behavior.

### **Hypothesis 2**

There is a positive relationship between inclusive climate and individual inclusive behavior.

## The Moderating Role of Inclusive Climate on the Prosocial Motivation-Inclusive Behavior Link

Team climate refers to the shared perceptions of the work environment in a group that make up the implicit rules which team members follow [39]. Accordingly, inclusive climate refers to the implicit rules team members adhere to about the way people with disabilities are accepted, helped and treated within the work team. Thus when inclusive climate for work groups is high, there will be a shared vision and set of rules on positive behavior that is acceptable and valued within the group, such as inclusive behavior. Both employees high and low in prosocial motivation, influenced by a high inclusive climate are consequently more likely to adhere to group norms and display more inclusive behavior. Therefore, inclusive climate might shape the expression of individual dispositions like prosocial motivation. Moreover, climates in general that aim to create positive environments have been argued to augment employees’ views on displaying citizenship behaviors [43].

In contrast, in work groups that rate the inclusive climate to be low, employees are likely to perceive that inclusive norms are less valued. In such a situation there is a reduced emphasis on inclusion toward people with disabilities and no external need to display inclusive behavior. However, as suggested earlier, based on the functional approach [9, 17, 30], one would expect those employees who are high in prosocial motivation to display inclusive behavior regardless of the external climate whereas this is less likely for employees with low prosocial motivation. We therefore hypothesize that inclusive climate interacts with prosocial motivation in predicting inclusive behavior.

### **Hypothesis 3**

Inclusive climate moderates the relationship between individual prosocial motivation and individual inclusive behavior, such that the relationship is stronger when inclusive climate is low and weaker when inclusive climate is high.

## Method

### Participants and Procedure

Respondents were 372 team members of 103 work teams from seven organizations, located throughout the Netherlands (response rate = 35 %). The organizations were active in e.g. the healthcare sector, super market industry, and the disability employment sector. The data used in this study was collected as part of a larger 4-year research project on inclusive organizations. Another publication that resulted from this project is Nelissen et al. [8] on how and when stereotypes relate to inclusive behavior toward people with disabilities.

Team members worked in teams with a minimum of three colleagues with the addition of one coworker with a disability (including various physical, cognitive, mental, sensory, and developmental impairments). All employees who work in teams with people with disabilities were provided with a personalized envelope containing two sets of questionnaires, one self-report questionnaire and one for their peers. All team members (except for the team member with a disability) filled in the inclusive team climate measure and provided self-ratings on prosocial motivation.

Individual inclusive behavior was assessed by peer-ratings, provided by 313 work colleagues (response rate 29.4 %). These peers were selected by the target participant, who was instructed to pair up with a colleague who knew him/her well, and regularly observed their daily work practices. Independently choosing a peer is common procedure to obtain reliable multiple source data [17].

The final sample consisted of data for 282 team members, distributed over 84 teams: Data for 35 participants was omitted because they worked in teams in which less than 3 members had responded; data for 55 participants could not be included in analyses because no peer-ratings of inclusive behavior were available. The average team size was 4.30 (*SD* = 1.41, ranging from 3 to 9 members/team). Jobs of team members entailed e.g. shelf re-stockers (42 %), nurses (10 %), or cashiers (8 %). Team members were 46 % male, with a total average tenure of 12.6 years (*SD* = 10.53), and 38 years of age (*SD* = 13.84).

### Measures

All measures, apart from the inclusive climate measure, were adopted from English and translated into Dutch with a translation and back-translation process, whilst taking into account the guidelines for test translation and adaptation [44]. All scales were assessed on a 5-point Likert scale ranging from 1 (strongly disagree) to 5 (strongly agree).

Individual team members provided ratings on prosocial motivation, and inclusive climate.

#### Prosocial Motivation

Participants completed the 5 items of the prosocial motivation measure put forward by Grant and Sumanth [45]. Sample items are “I prefer to work on tasks that allow me to have a positive impact on others”, “It is important to me to have the opportunity to use my abilities to benefit others”. Chronbach’s Alpha was *α* = .86.

#### Inclusive Climate

Since, to our knowledge, a measure of inclusive climate has not yet been proposed in the literature, we developed an inclusive climate measure. It consisted of 5 items, mirroring the definition of inclusive climate presented above: “In my team people with disabilities are accepted,” “In my team people with disabilities are helped,” “In my team people with disabilities are treated as other colleagues,” “In my team we are attentive to the needs of people with disabilities”, and “In my team we are attentive to the opportunities of people with disabilities” (*α* = .90). We calculated both within-group agreement [rwg(j)] and intra class coefficients (ICC) to provide empirical justification for aggregating data to the team level [46]. The mean rwg(j) of inclusive climate was .85. Following LeBreton and Senter [46] values lying between .71 and .90 indicate strong agreement among raters. Furthermore, analyses revealed an ICC1 value of .18, and ICC2 value of .43. ICC1 values lying between .10 and .25 indicate a medium to strong effect, justifying aggregation to the team level [46].

Peers (work colleagues) provided ratings on the target person’s inclusive behavior.

#### Inclusive Behavior

We assessed inclusive behavior with an 8-item scale adapted from the altruism and courtesy subscales of a measure of organizational citizenship behavior [8, 22]. The scales were adapted to the viewpoint of the participant: peers’ questionnaires referred to their colleague (*α* = .89). The questions were; “My colleague does not abuse the right of people with disabilities”, “My colleague tries to avoid creating problems with people with disabilities”, “My colleague considers the impact of his/her actions on people with disabilities”, ”My colleague helps people with disabilities who have been absent”, “My colleague helps people with disabilities who have heavy workloads”, “My colleague helps orient new people with disabilities even though it is not required”, “My colleague willingly helps people with disabilities who have work related problems”, “My colleague is always ready to lend a helping hand to people with disabilities around him/her”.

### Statistical Analysis

We conducted multilevel random coefficient modeling following Bliese [47], using the nlme package (linear and nonlinear mixed effect models; [48] and the multilevel [49] package in the R environment (R Core Team 2012). Multilevel random coefficient modeling is a statistical procedure developed for testing hierarchically nested data structures, such as ours where employees (level 1) are nested in work teams (level 2). Predictor variables at both levels were grand mean centered following recommendations to base centering decisions on theoretical considerations [50, 51]. Our theoretical argumentation does not suggest a frog-pond model (in which researchers are interested in deviations from the team average), but rather suggests that absolute levels of prosocial motivation are related to inclusive behavior. Accordingly, we grand-mean-centered level 1 variables.

## Results

Means, standard deviations, and intercorrelations between study variables are displayed in Table 1. To test Hypotheses 1 and 2, we conducted a multilevel analysis predicting individual inclusive behavior from individual prosocial motivation and team inclusive climate (see Table 2, Model 1). Results revealed that individual prosocial motivation was positively related to individual inclusive behavior (estimate = .21, *p* < .001), supporting Hypothesis 1. Furthermore, in line with Hypothesis 2, inclusive team climate was significantly related to individual inclusive behavior (estimate = .51, *p* < .001).

**Table 1:** Means (M), standard deviations (SD), reliability estimates (α) and intercorrelations of study variables

Variable		*n*	*M*	*SD*	α	1	2	3
Individual level								
1. Prosocial motivation		279	4.07	.59	.86	–		
2. Individual inclusive behavior		282	4.06	.61	.89	.28**	–	
Team level								
3. Inclusive climate		84	3.97	.77	.90	.43**	.38**	–

Means, standard deviations, and reliability estimates are individual level

** *p* < .01

**Table 2:** Multilevel model predicting individual inclusive behavior

	Model 1			Model 2			Model 3		
	Coef.	*SE*	*t*	Coef.	*SE*	*t*	Coef.	*SE*	*t*
Fixed effects									
Intercept	4.02	.04	104.20***	4.04	.04	106.52***	4.05	.04	150.83***
Inclusive climate (IC)	.47	.09	5.34***	.41	.08	4.92***	.45	.08	5.30***
Prosocial motivation (PM)	.17	.06	2.99**	.19	.07	2.82**	.21	.06	3.40***
IC × PM							−0.27	0.13	−2.16*
Random effects									
Intercept SD		.19			.18			.17	
Residual SD		.52			.50			.51	
Prosocial motivation (PM) SD					.26			.15	

Model 1 = fixed slope model; Model 2 = random slope model; Model 3 = random slope model with interaction term

* *p* < .05; ** *p* < .01; *** *p* < .001

**Fig. 2:**
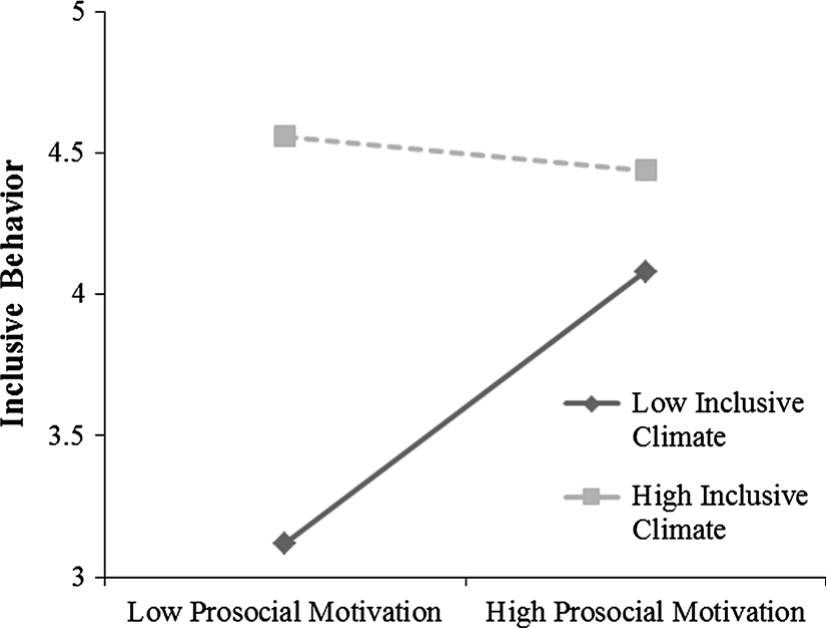
Interaction of inclusive climate and prosocial motivation on individual inclusive behavior

To test the moderating role of team inclusive climate (Hypothesis 3), we followed the procedure described in Bliese [47]. Accordingly, we added a random slope for prosocial motivation in Model 2. We then investigated whether Model 2 provided a better fit to the data than Model 1. Model comparisons were conducted using the anova function provided in the nlme package which tests differences in model deviances (using −2log likelihood values) between both models based on a Chi-square distribution [47]. Although −2 log likelihood values were slightly lower for Model 2 compared to Model 1, the difference was not statistically significant [χ^2^(2) = 3.73, *p* > .05]. However, due to the low power of such tests, researchers have strongly recommended testing theoretically hypothesized cross-level interactions regardless of significance of slope variance (as estimated with likelihood ratio tests [52]. We therefore proceeded to test whether team inclusive climate interacted with prosocial motivation in predicting inclusive behavior (Hypothesis 3). Accordingly, Model 3 revealed a significant interaction between prosocial motivation and team inclusive climate (estimate = −0.27, *p* < .05), supporting Hypothesis 3.^1^ Simple slopes analyses revealed a significant relation between prosocial motivation and inclusive behavior when inclusive climate was low (*β* = .49, *p* < .01), and no significant relationship when inclusive climate was high (*β* = −.06, n.s). A graphical depiction of this interaction effect is shown in Fig. 2.

## Discussion

In response to recent calls in the literature that prosocial motivation might have broader social implications with regards to corporate social responsibility [28], this study set out to investigate the relationship of prosocial motivation and helping behavior directed at people with disabilities, referred to as inclusive behavior. We furthermore aimed to extend the multilevel literature on team level contextual variables that influence individual level relationships, by showing that the inclusive climate might be an important boundary condition for the display of inclusive behavior. Specifically, our study shows that prosocial motivation is positively related to individual inclusive behavior, as has been previously found in relation to helping behavior in general [27]. The confirmation of Hypothesis 1 validates our premise that, from the perspective of the functional approach, people might try to satisfy their needs and goals by displaying inclusive behavior.

Furthermore, our study demonstrates that climate is an important contextual variable that has both direct and indirect effects on individual inclusive behavior. In particular when people with disabilities are concerned, colleagues showed more inclusive behavior when the group had an inclusive climate. More specifically, Hypotheses 2 and 3 were confirmed and we found that inclusive climate is not only directly related to individual inclusive behavior, but that it also moderates the relationship between prosocial motivation and individual inclusive behavior. This interaction reveals that a high inclusive climate as a team level variable seems to be strong enough to shape the individual’s prosocial motivation. This shows that certain situations can be strong enough to restrain individual characteristics, because employees will adhere to the group settings as long as the norms are perceived in a collectivistic manner. These findings highlight the importance of a multilevel approach, because team level variables might show to have an overarching effect on individual inclusive behavior, which might not have been revealed using a single level approach.

From a theoretical point of view, our study aims to extend the understanding of contextual variables, such as inclusive climate, as multilevel occurrences, which have rarely been investigated in relation to helping behavior. Our research adds to current knowledge on helping behavior by showing that the relationship between prosocial motivation on the one hand, and helping behavior on the other hand, is not only confirmed, but also encompasses the specific kind of helping behavior towards people with disabilities. Furthermore, our results may contribute to previous qualitative studies in acknowledging the importance of the way people with disabilities are treated in the workplace [53] and may pertain to aid the organizational socialization process which has beneficial effects on performance, job satisfaction and even turnover intentions of people with disabilities [54]. Additionally, in general terms, characteristics of the workplace at the departmental and individual level have been put forward as being important to the inclusion and participation level of people with disabilities [55]. Therefore, our findings on team inclusive climate and individual prosocial motivation can provide insight in factors that contribute to the workplace inclusion of people with disabilities, thereby serving the goal of this study.

Finally, our study contributes to the emerging field of corporate social responsibility; this research meets the call for new directions in IO Psychology by Colella and Bruyère [5] to address the gap in literature on what happens to people with disabilities once they enter the labor market. However, with a focus on factors that contribute to the inclusion of people with disabilities seen from a multilevel perspective. This new direction can be seen as an important issue to researchers, as the work-life journey of people with disabilities only begins when they find work.

## Limitations, Strengths, and Directions for Future Research

Our study has some limitations that should be considered in future research. First, our results are based on cross-sectional data. We can therefore not draw any causal inferences based from our data and causal pathways may also be reversed or reciprocal. Although theory suggests, that causal pathways are such that prosocial motivation and inclusive climate precede inclusive behavior, we cannot rule out that, for instance, inclusive behavior also influences inclusive climate. In our situation, it is however not reasonable to assume that the ratings of peers would have an influence on the behavior of the employees. The displayed behavior of employees, on the other hand, should have an effect on the ratings of their peers. Future research may therefore benefit from investigating relationships, ideally with a cross-lagged panel design, allowing to investigate reverse and reciprocal causation.

Second, the relatively low response rate of 35 and 29.4 % for employees and their peers, respectively, might give rise to a non-response bias. However, when examining the response rates in more detail, it shows that the low number of returned questionnaires is mainly due to one organization. This organization had the potential of providing many work teams but was still in a pilot project phase, a situation which could explain the lower response rate. The other six organizations provided a normal response rate of 58.9 and 53.8 %, respectively [56].

Thirdly, employees worked with employees that have a large variety of disabilities, encompassing physical, cognitive, mental, sensory, and developmental disabilities. Since employees may react differently to people with different sorts of disabilities, effects of type of disability both as a predictor of inclusive behavior as well as a moderator, may also be investigated in future research. In addition, inclusive behavior was assessed, using a questionnaire, whereas observations of actual behavior at the workplace by independent observers rather than work colleagues would have obtained data that may be less susceptible to social desirable responding. However, a notable strength of this study is the multiple source data we used (predictors assessed by team members and inclusive behavior by work colleagues) allowing for independent assessments of inclusive behavior and subsequently the reduction of common method bias [57].

Our study provided valuable first insights into the factors that contribute to the inclusion of people with disabilities once they have entered the labor market. More empirical research is needed to address the aspects that might influence the work situation of people with disabilities. Moreover, with regard to inclusive behavior, relationships to performance, productivity, well-being, as well as the opinions of people with disabilities on these matters, need to be addressed in future research; in order to make sure that inclusive behavior does indeed lead to a better integration and more sustainable employment for people with disabilities.

## Practical Implications

Inclusive behavior and inclusive climate are conceptualized as prerequisites for the boundary conditions of inclusion for people with disabilities, and are generally sought after in inclusive organizations that value a diverse workforce [58, 59]. Whereas previous studies have focused on the organizational socialization and the need for external and internal support for people with disabilities [54], we argue that the prevalence of inclusive behavior might help to attain inclusion for people with disabilities in inclusive organizations. Inclusion of people with disabilities in their team is deemed to be an important factor for success in the workplace [5].

This study shows that inclusive organizations need to be aware of not only individual employee characteristics, but also team level climate to ensure the smooth integrations of people with disabilities into regular work teams. These findings may provide opportunities for organizations to become more inclusive, and attract a more diverse workforce. It is hard to change employees’ individual mindset or motivation, but it is actually feasible to foster the inclusive climate. By expressing an inclusive mindset at an organizational level, but also through educating team leaders and supervisors to iterate the inclusive message, climates can be shaped. Supervisors have a strong hand in transforming their teams by leading by example in norms and values, which fit the general criteria of a climate.

Fostering sustainable employment for employees with disabilities might be a first step to address some of today’s society major issues. Future employment levels are decreasing because the baby-boom generation has reached the age of retirement, whilst on the other hand, several groups, such as people with disabilities, are not considered to participate in the labor force [58]. Thus, seeking to integrate people with a broad range of disabilities more fully into the workforce might counter the unemployment rates, and signify a cut back on welfare payments, but especially allow people with disabilities to fully participate in our society.

## Footnotes

1 Since some authors have recommended group mean centering when testing cross-level interactions [50, 60], we ran the same set of analyses group-mean-centering prosocial motivation. The same pattern of results emerged, although the cross-level interaction was somewhat weaker (*p* = .10).
